# Modulation of the Bifidobacterial Communities of the Dog Microbiota by Zeolite

**DOI:** 10.3389/fmicb.2016.01491

**Published:** 2016-09-22

**Authors:** Alberto Sabbioni, Chiara Ferrario, Christian Milani, Leonardo Mancabelli, Enzo Riccardi, Francesco Di Ianni, Valentino Beretti, Paola Superchi, Maria C. Ossiprandi

**Affiliations:** ^1^Department of Veterinary Medical Science, University of ParmaParma,Italy; ^2^Laboratory of Probiogenomics, Department of Life Sciences, University of ParmaParma, Italy

**Keywords:** dogs, gut microbiota, chabazitic zeolitite, *Bifidobacterium*, adsorptive capacity

## Abstract

During last decades canine health and well being is becoming an important issue for human owners. In dogs, several factors including diet, pathogenic bacterial and stress conditions can affect the composition of the gut microbiota. In this study, we evaluated the effect of dietary chabazitic zeolitite (CZ) supplementation on the contribution of bifidobacteria to the fecal microbiota in training hunting dogs. Fecal microbiota cataloging based on 16S rRNA microbial profiling analyses highlighted an increase of *Lactobacillus* and *Bifidobacterium* in animals treated with CZ, with a simultaneous decrease of pathogens associated with dog gastrointestinal infections, such as *Klebsiella* and *Enterobacter*. A detailed profiling of the bifidobacterial population of dogs receiving CZ based on the ITS-based sequencing approach, revealed an enhancement bifidobacterial of species typical of animals such as *Bifidobacterium animalis* and *B. pseudolongum*. Moreover, these analyses identified the occurrence of putative new bifidobacterial taxa in both treated and untreated samples.

## Introduction

Pet population is increasing in western countries, and dogs are the major human companions. Mutual interest has evolved into companion animals being a stable part of human life and therefore, the health and wellbeing of pets have increasingly raised interest during last decades. During history, the dog diet has changed, starting from a carnivorous behavior and a high protein diet (Clauss et al., 2010) to a carbohydrate rich diet and an urban life-style.

Despite the long span history of human-dog co-evolution, the knowledge of canine intestinal microbiota composition is much less complete than for humans. The dog gastro-intestinal tract (GIT) represents a rich ecosystem, composed of a wide range of metabolically active microorganisms (Simpson et al., 2002; Suchodolski et al., 2008; Kerr et al., 2013a). The predominant bacterial phyla in the colon and faeces of dogs are represented by *Firmicutes* (40–60%), *Bacteroidetes* (5–10%), *Proteobacteria* (15–20%), and *Fusobacteria* (5%) (Kerr et al., 2013b; Deng and Swanson, 2015), representing approximately 99% of the gut microbiota in dogs. However, very little is known about the occurrence of healthy promoting microorganisms such as bifidobacteria in the gut especially using metagenomics based approaches (Gavini et al., 2006; Jia et al., 2010).

Bifidobacteria are Gram positive bacteria that colonize different ecological niches, but represents one of the dominant colonizers of mammals at the very early first stages of life (Milani et al., 2015). The analyses of the gut microbiota of different mammals indicate that some bifidobacterial species, usually detected in the human GIT, were also identified in many other animals (Lamendella et al., 2008). For example, *Bifidobacterium bifidum, B. adolescentis, B. catenulatum*, and *B. dentium* are human-type bifidobacteria (Duranti et al., 2015, 2016), but these taxa displayed a cosmopolitan ecological behavior among different mammals (Lamendella et al., 2008).

In hunting dogs, emotional stress to which they are submitted during the training, can alter the habitat of the GIT (Rutgers et al., 1996). Therefore, to keep a suitable function of the GIT through appropriate feeding strategies is interesting, to avoid the intestinal colonization by enteropathogens (e.g., *Escherichia coli, Salmonella* ssp., *Clostridium perfringens, C. difficile*) (McKenzie et al., 2010; Kerr et al., 2013a).

To avoid antibiotic therapies, alternative products are under investigation. Zeolitites are aluminosilicates characterized by an open structure, which can accommodate a wide variety of ions. The particle size, crystallite size, and the degree of aggregation of the zeolitic material, as well as the porosity of individual particles, determine the access of ingesta fluids to the zeolitic surface during the passage across the GIT, and strongly affect its ion exchange, adsorption and catalytic properties (Papaioannou et al., 2005). The mechanism of action of zeolite is likely to be multifunctional. Different health and performance promoting properties were highlighted for zeolite in animal diet. These include ammonia binding effect, fecal elimination of *p*-cresol, retarding effect on digesta transit, enhanced pancreatic ezymes activity, and aflatoxin sequestering effect (Papaioannou et al., 2005). Moreover, recently it was reported the application of zeolite in reducing pathogens counts in broiler chicken (Prasai et al., 2016). Among zeolitites, the chabazitic zeolitite (CZ) has a high cation-exchange capacity and bulk density (Pabalan and Bertetti, 2001). Dietary inclusion of zeolitites has been effective in animals (e.g., pigs, calves) and humans suffering from gastrointestinal disturbances (RodriguezFuentes et al., 1997; Papaioannou et al., 2005). To date, no data exist about the evaluation of the effects of zeolitites on dog intestinal microbiota. The aim of the present study was to assess the effect of dietary CZ supplementation on the fecal microbiota with particular emphasis on bifidobacterial populations in training hunting dogs through culture-dependent methods and 16S rRNA/ITS (internal transcribed spacer) microbial profiling approach.

## Materials and Methods

### Ethics Statement

This study was carried out in accordance with the recommendations of the ethical committee of the University of Parma. The protocol was approved by the “Comitato di Etica Università degli Studi di Parma”, Italy. All animal procedures were performed according to national guidelines (Decreto legislativo 26/2014) on the protection of animals used for scientific purposes.

### Animals and Experimental Procedure

Twenty adult English Setter dogs, reared in the same kennel, were selected to be homogeneous with reference to age (mean age ± SD: 3.50 ± 1.9 years), body weight (mean weight ± SD: 18.83 ± 2.96 kg) and gender (10 males, 10 not pregnant females). Based on age, weight, and sex animals were equally divided into two groups (10 dogs group^-1^), individually penned with a rest area inside (2.70 m× 1.40 m) and a paddock outside (4.50 m× 1.40 m). Animals were free of any clinical symptoms indicating gastrointestinal disease and they did not receive medications that are expected to alter the gut microbiota such as antibiotics. Dogs were wormed one month before the start of study. The characteristics of the groups are reported in **Table 1**. During a period of 28 days, both groups received a diet, based on raw poultry meat (25% crude protein, 24% ether extract, 5% ash, 2% crude fiber, and 18.4 MJ kg^-1^ ME, on dry matter). The individual ration, administered at about 25 g dry matter kg^-1^ of body weight^0.75^, once a day, was supplemented (group Tr) or not (group NTr) with CZ powder at the dose of 5 g day^-1^. For each dog, zeolitite was weighed and added to the ration at each meal. Free access to water was provided. During the study, all dogs were daily subjected to an aerobic physical activity characterized by gallop for 20 min, according to the trainer’s practices. Training was performed in two outdoor next areas, at a mean temperature and relative humidity of 24 ± 3°C and of 67 ± 10%, respectively. Inside each group, five pairs of dogs were identified and each of them assigned alternatively to one or to the other of the training areas.

**Table 1:** Characteristics of the experimental groups (mean ± SD).

Parameter	Groups^∗^	
	NTr	Tr
Animals (No.)	10	10
Age (years)	3.41 ± 1.59	3.50 ± 1.60
Body weight (kg)	19.59 ± 2.85	18.08 ± 2.74

*^∗^NTr, untreated group; Tr, treated group.*

### Chabazitic Zeolitite Source

The powder of CZ, was obtained after sterilization at 200°C for 20 min (Chabasite 70^®^ Verdi S.p.A, Italy). The total zeolitic content was 70 ± 5%, of which 65 ± 3% due to chabazite (Na0.14K1.03Ca1.00Mg0.17) [Al3.46Si8.53O24] × 9.7H_2_O and 5 ± 3% to phillipsite (Na0.9Ca0.5K0.6) [Si5.2A12.8O16] × 6H_2_O. No traces of clinoptilolite were found. The composition of zeolitic powder was determined by Rietveld-RIR method (Gualtieri, 2000). The cation-exchange capacity and bulk density in relation to particles size were 2.2 ± 0.1 mEq g^-1^ and 0.70-0.90 g (cm^3^)^-1^, respectively (Gualtieri, 2000; Cresswell and Hamilton, 2002). Water retention in relation to particles size was about 30-40% (w/w). The granulometry of the powder was less than 100 μm.

### Collection of Fecal Samples

Feces consistency was scored using a scale of 1 (hard) to 5 (watery) (Grieshop et al., 2002) at days 0 (Time point 0, T0), 16 (Time point 1, T1) from the beginning of the dietary treatment, and at the end of experimental period (day 29, Time point 2, T2). During the same days, individual fecal samples were collected directly from the rectum, using a sterile glove lubricated with water. The feces were placed in sterile polyethylene bags, immediately transported to the laboratory on ice packs and frozen at -20°C until analysis.

### 16S rRNA/ITS Microbial Profiling

Upon arrival at the laboratory, individual fecal samples were aliquoted and combined with other individual samples from the same treatment to form pooled samples. In fact, in animal health it has been shown recently that pooling stool samples allows a rapid assessment of infection intensity and drug efficacy (Mekonnen et al., 2013). Each individual dog sample was equally represented in the respective pooled sample. DNA was extracted from pooled fecal samples using the QIAamp DNA Stool Mini kit following the manufacturer’s instructions (Qiagen Ltd., Strasse, Germany).

Partial 16S rRNA gene sequences were amplified from extracted DNA using primer pair Probio_Uni and /Probio_Rev, which target the V3 region of the 16S rRNA gene sequence, as previously reported (Milani et al., 2013). Partial ITS sequences were amplified from extracted DNA using the primer pair Probio-bif_Uni/Probiobif_Rev as described by Milani et al. (2014b). The PCR conditions used were 5 min at 95°C and 35 cycles of 30 s at 94°C, 30 s at 55°C, and 90 s at 72°C, followed by 10 min at 72°C. Amplification was carried out using a Veriti Thermocycler (Applied Bio-systems).

16S rRNA gene and ITS sequencing were performed using a MiSeq (Illumina) according to the protocols previously published (Milani et al., 2013, 2014b).

### 16S rRNA Gene-Based Microbiota Analysis

The achieved individual sequence reads were filtered by the Illumina software to remove low quality and polyclonal sequences. All Illumina quality-approved, trimmed, and filtered data were exported as.fastq files. The.fastq files were processed using a custom script based on the QIIME software suite (Caporaso et al., 2010). Paired-end reads pairs were assembled to reconstruct the complete Probio_Uni/Probio_Rev amplicons. Quality control retained sequences with a length between 140 and 400 bp and mean sequence quality score >20 while sequences with homopolymers >7 bp and mismatched primers were omitted. In order to calculate downstream diversity measures (alpha diversity indices, Unifrac analysis), 16S rRNA Operational Taxonomic Units (OTUs) were defined at ≥97 % sequence homology using uclust (Edgar, 2010) and OTUs with less than 10 sequences were filtered. All reads were classified to the lowest possible taxonomic rank using QIIME (Caporaso et al., 2010) and a reference dataset from the SILVA database (Quast et al., 2013). Biodiversity of the samples (alpha-diversity) were calculated with Chao1 index.

### ITS-Based Microbiota Analysis

For ITS-based microbiota analysis Fastq files obtained by sequencing of the ITS amplicons were analyzed using a custom script, named bif_ITS_analysis.sh script ^1^. This script requires QIIME (Caporaso et al., 2010) to be installed (or works in a QIIME virtual machine) and accepts.bam or.fastq input files containing sequencing reads. Input data were processed as previously described (Milani et al., 2014b).

### Bacterial Counts

The homogenates fecal specimens were serially diluted with both half-strength Wilkins-Chalgren Anaerobe Broth (WCAB) and Buffered Peptone Water (ThermoScientific-Oxoid, UK). Dilutions in duplicate were plated on MacConkey agar (Merck, Germany) for Enterobacteriaceae, Perfringens agar Base (OPSP) (Oxoid, UK) for *C. perfringens*, vancomycin and bromocresol green (LAMVAB) agar (Hartemink and Rombouts, 1999) for lactobacilli, and Azide maltose agar (Biolife, Italy) for enterococci counts. MacConkey agar and Azide maltose agar plates were incubated aerobically at 37°C for 24 and 48 h, respectively. Other media were incubated anaerobically at 37°C for 48-72 h. The taxonomy of colonies isolated random on selective media were determined at genus and species level by API System (Bio-Merieux, Italy) to verify the reliability of the media utilized.

### *In vitro* CZ Adsorptive Capacity

The ability of CZ to bind to enteropathogens bacteria was evaluated in pooled feces using two reference strains, i.e., *E. coli* ATCC 35218 and *C. perfringens* ATCC 13124. Strains were grown in Mueller-Hinton Broth (Difco, MI, USA) at 37°C for 24 h, then transferred to 10 ml of broth and grown for another 8 h to reach the final exponential phase.

Adsorptive capacity of CZ was evaluated, measuring spectrophotometrically the OD of the samples (An and Friedman, 1997). Twenty-five grams of pooled feces obtained by NTr groups and collected on days 0, 16, and 29 were placed, in triplicate, into flasks containing 225 ml of Buffered Sodium Chloride-Peptone Solution pH 7.0 (Oxoid, UK). CZ was added in different quantities (0, 0.25, 0.5, 1 g). Lastly, *C. perfringens* ATCC 13124 or *E. coli* ATCC 35218 strains were added to medium and incubated at 37°C. At 0, 2, 4, 6, and 24 h, 150 μl of the suspension were transferred into a microtiter plate in four replicates and the absorbance was immediately evaluated (VICTOR3, 1420 multilabel counter, PerkinElmer, Italy) at 620 nm.

### Statistical Analysis

Data for fecal score and fecal bacteria counts were checked for normality and then analyzed by ANOVA using the GLM procedure in SAS (Version 9.4, SAS Institute Inc., USA). The mixed model included the fixed effects of group (two levels), of sampling time (three levels), the interaction between group and sampling time and the random effect of animal. Values of colony forming units (CFU) have been expressed as log_10_ g^-1^ of feces.

Statistical significance was reached for *P* ≤ 0.05 as a *P*-value >0.05 and ≤0.10 was considered as a trend.

### Data Deposition

Raw sequences of 16S rRNA gene profiling are accessible through SRA study accession number SRP075756. Raw sequences of ITS profiling are accessible through SRA study accession number SRP080281.

## Results

### 16S rRNA Profiling of CZ Treated Dog

Pooled fecal samples from CZ treated (Tr) and no-treated (NTr) dogs were obtained in order to assess the microbiota composition based on 16S rRNA-sequencing analysis as described previously (Milani et al., 2013). The sequencing produced a total of 589784 reads with an average of 98297 reads per sample (Supplementary Table S1).

**FIGURE 1:**
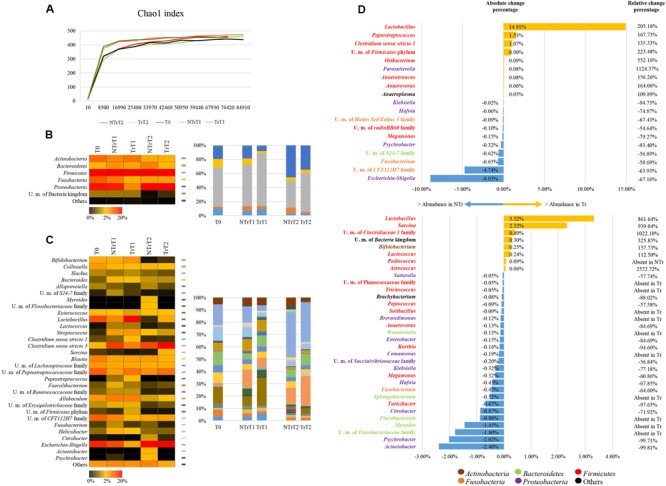
**Exploration of the taxonomic profile of NTr and Tr groups. (A)** Shows the rarefaction curves representing variation of the Chao1 and the Shannon diversity indexes at increasing sequencing depth of NTr and Tr fecal samples. **(B)** Displays bar plots and heat map of the identified bacterial phyla in the pooled CZ treated or untreated samples. **(C)** Represents bar plots and heat map of the identified bacterial genera in the pooled Tr or NTr samples. **(D)** exhibits the variation of taxa at time point T1 (upward) and T2 (below). We reported the bacterial genera with absolute change percentage >0.05% and showing increase >100% or decrease <-50% of relative change percentage in Tr data sets as compared to those obtained from NTr samples. In all panels the term unclassified member is abbreviated to U. m..

Assessment of rarefaction curves, based on the Chao1 biodiversity indexes calculated for 10 subsampling of sequenced read pools, indicated that both curves tend to reach a plateau. Therefore, in all cases the obtained sequencing data was deemed adequate to cover the vast majority of the biodiversity contained within the samples (**Figure 1A**). Moreover, the two curves did not show relevant differences, thus indicating that the analyzed samples have similar biodiversity.

### Gut Microbiota Composition of CZ Treated Dogs

During the study, diarrhea events were not observed in the CZ treated dogs. CZ did not affect the palatability of the feed, which was eaten completely within 30 min after dosing. Fecal scores were not affected by the factors in the statistical model (*P* > 0.05; **Table 2**). Fecal microbiota differences were observed in relation to group and sampling time (*P* < 0.05).

**Table 2:** Effects of chabazitic zeolitite (CZ) supplementation on fecal score and fecal microbial concentration (least squares means of log_10_ CFU g^-1^ of feces).

Parameter	Groups^∗^		Sampling time			SEM^†^	*P*-values		
	NT	T	0	T1	T2		G^‡^	St^‡^	GxSt^‡^
Faecal score^§^	3.15	3.35	3.07	3.33	3.37	1.18	NS	NS	NS
*Lactobacillus* ssp.	7.22	7.59	7.18	7.33	7.72	0.08	<0.001	<0.001	<0.001
*Enterococcus* ssp.	7.19	7.51	7.15	7.22	7.68	0.05	<0.001	<0.001	<0.001
*Enterobacteriaceae*	7.18	6.85	7.17	7.19	6.69	0.06	<0.001	<0.001	<0.001
*Clostridium perfringens*	7.36	6.99	8.18	6.64	6.71	1.27	NS	NS	NS

*^∗^NTr, untreated group; Tr, treated group. ^‡^SEM, standard error of the difference of means. ^‡^G, group effect; St, sampling time effect; G × St, interaction. ^§^ On a scale of 1 (hard) to 5 (watery).*

Inspection of predicted taxonomic profiles at phylum level for all NTr samples (T0, NTrT1, NTrT2) highlighted that *Firmicutes* (average 51.15% ± 11.46%) represented the dominant phylum of the cecal community in dogs, outnumbering the *Proteobacteria* (average 27.06% ± 15.75%), the *Fusobacteria* (average 8.54% ± 3.46%) and the *Bacteroidetes* (average 5.49% ± 2.62%) phyla (**Figures 1B,C**).

The comparison of the average relative abundance of NTr and Tr samples at time point T1 revealed a decrease of members of the *Enterobacteriaceae* family (-66.99 %), such as *Escherichia* (-67.16%), *Klebsiella* (-94.75%), and *Hafnia* (-74.87%), in Tr samples (**Figure 1D**) and an increase of *Lactobacillus* (205.16%) and *Bifidobacterium* (75.35%) in CZ treated animals (**Figure 1D**). At time point T2 in CZ treated animals (**Figure 1D**), the decrease in *Enterobacteriaceae* (-15.34%), includes a reduction of the genera *Hafnia* (-67.85 %), *Klebsiella* (-77.18%), and *Enterobacter* (-84.69%), along with an increase in relative abundance of *Lactobacillus* (861.64%) and *Bifidobacterium* (157.73%) (**Figure 1D**).

Notably, data achieved with culture-dependent approaches largely confirmed results obtained with 16S rRNA microbial profiling. In fact, *Lactobacillus* ssp. and *Enterococcus* ssp. counts were higher, while *Enterobacteriaceae* counts were lower in Tr than in NTr group (*P* < 0.05). *Lactobacillus* ssp. counts tended to be higher in Tr than in NTr group on day 16 (T1; 7.43 vs. 7.24; *P* < 0.10) and were higher on day 29 (T2; 8.18 vs. 7.25; *P* < 0.05). An increase of *Enterococcus* ssp. concentration (8.10 vs. 7.27) and a decrease of *Enterobacteriaceae* counts (6.24 vs. 7.14) were found in Tr compared to NTr group on day 29 (T2; *P* < 0.05). Besides, no change on the fecal *C. perfringens* counts was reported in relation both to the sampling time and to the treatment (*P* > 0.05).

### Bifidobacterial Community Modulation by CZ

Focusing on the contribution of bifidobacteria to the overall dog microbiota, it is worth noticing that at day 0 (T0) and in NTr animals at days 16 and 29 (T1 and T2, respectively), this genus represents 2.32% ± 1.88% of the gut microbiota of hunting dogs. In treated animals (Tr) the presence of the *Bifidobacterium* genus showed an increase of about 157.73% compared with Tr animals at T2, after 29 days of CZ diet (**Figure 1D**).

In order to precisely catalog the effects on the bifidobacterial population of dogs after CZ treatment, we performed an ITS profiling of bifidobacterial communities in stool samples of Tr and NTr dogs.

Quality filtering of the sequenced ITS amplicons produced an average of 52468 high-quality and full-length reads per sample (Supplementary Table S2) that were taxonomically attributed reaching the minimal taxonomical rank of species.

**FIGURE 2:**
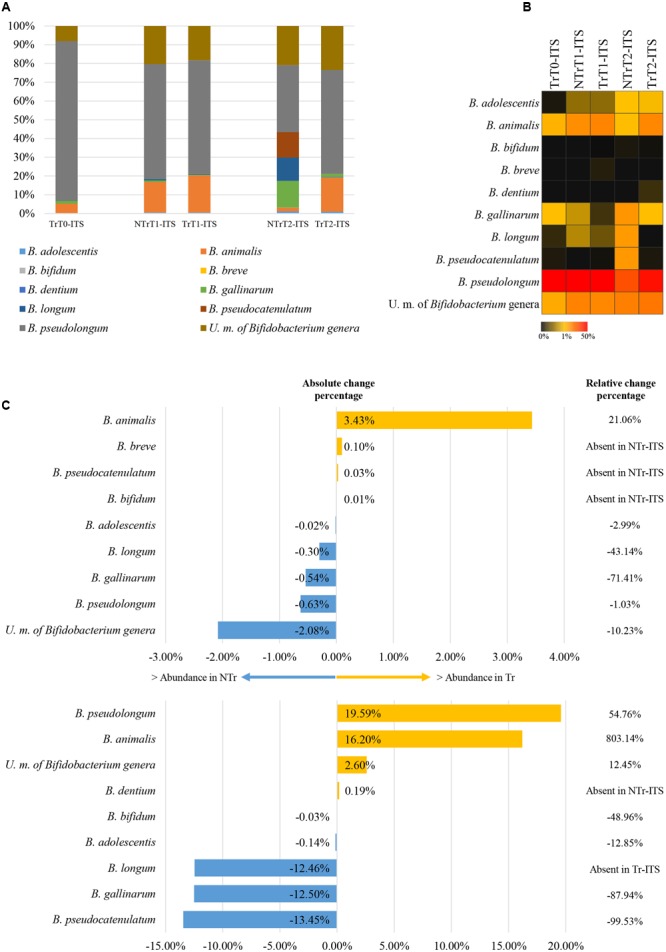
**Exploration of the bifidobacterial population of NTr and Tr groups. (A)** Represents the bar plots of the identified bifidobacteria in the pooled CZ treated or untreated samples through the ITS analysis. **(B)** Shows heat map of the identified bifidobacteria in the pooled Tr-ITS or NTr-ITS samples. **(C)** Displays the variation of the bifidobacterial population at time point T1 (upward) and T2 (below). We reported the *Bifidobacterium* species with absolute change percentage >0.05 % and showing increase >100% or decrease <-50% of relative change percentage in Tr-ITS data sets as compared to those obtained from NTr-ITS samples. In all panels the term unclassified member is abbreviated to U. m..

The composition of bifidobacterial populations of dogs included in the analysis showed the presence of peculiar species, such as *B. pseudolongum* (average of 60.70% ± 24.61% for T0 and NTr samples) and *B. animalis* (average of 7.84% ± 7.50% in T0 and NTr dogs) (**Figure 2A**), which have been previously described to be typical of the animal GIT (Milani et al., 2014a) and especially of the dog GIT (Gavini et al., 2006). Notably, other bifidobacterial species previously described to be typical of the human gut such *B. catenulatum* and *B. bifidum* were detected at a lower extend (**Figure 2B**).

Furthermore, in untreated animal samples (NTr1 and NTr2), ITS analysis revealed the occurrence of *B. longum, B. gallinarum*, and *B. pseudocatenulatum* species, typical human bifidobacterial taxa (Milani et al., 2014a). One possible explanation of the presence of these species in the canine gut microbiota could be a bacterial transmission between animals and trainers as previously reported in literature (Song et al., 2013). However, further investigations will be needed. Notably, a large proportion of the OTUs defined as ‘unclassified’ in T0 dog samples (**Figures 2A,B**) clusters separately from any current known bifidobacterial taxon, thus putatively representing novel *Bifidobacterium* taxa. These putative new unclassified bifidobacterial species represents the second most present bifidobacterial taxa in the dog microbiota, in both Tr and NTr animals (**Figures 2A,B**).

As reported above, at time point T2 in CZ treated animals, there was an increase in relative abundance of the genus *Bifidobacterium* (**Figure 1D**). ITS profiling experiments revealed an increase of 803.14 and 54.76% of *B. animalis* species and *B. pseudolongum* species, respectively, after the addition of CZ. Moreover, a slight increase was detected also for the here identified putative new bifidobacterial taxa in TrT2 (12.45 %) compared to NTrT2 (**Figure 2C**).

### *In Vitro* Bacterial Adsorptive Test

**FIGURE 3:**
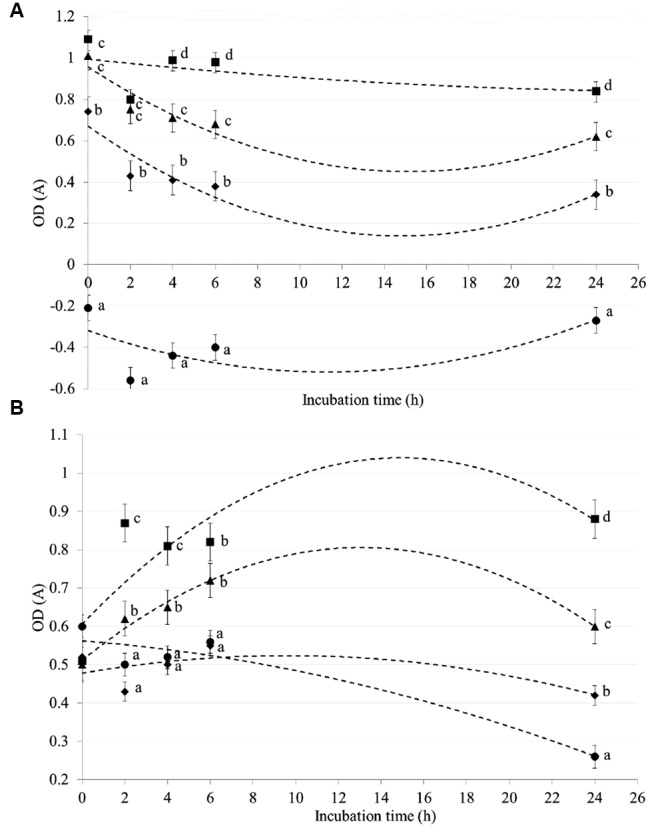
***In vitro* adsorptive capacity of chabazitic zeolitite (CZ) toward *Escherichia coli***(A)** and *Clostridium perfringens***(B)**.** CZ levels: (▪) 0 g, (▲) 0.25 g, (◆) 0.5 g, (●) 1 g. Error bars indicate standard errors; a, b, c, d, *P* < 0.05 (differences among CZ levels).

CZ showed an adsorptive capacity toward *E. coli* (**Figure 3A**) and *C. perfringens* (**Figure 3B**) strains in a dose- and time-dependent trial. Differences among CZ levels were registered for both strains after 2, 4, 6, and 24 h of incubation (*P* < 0.05). In particular, higher adsorptive capability against *E. coli* strain, was observed when CZ was added to the medium at a dose of 0.5 and 1 g rather than of 0 and 0.25 g (*P* < 0.05). When CZ was added at a dose of 1 g, negative values of OD starting from 0 h of incubation was observed for *E. coli*. During the first six hours of incubation, the adsorptive effect of CZ on *C. perfringens* strain was higher for levels of 0.5 and 1 g, than of 0 and 0.25 g (*P* < 0.05; **Figure 3**).

## Discussion

In hunting dogs, emotional factors, such as those to which they are submitted during the training, can affect the GIT permeability, motility, secretion and mucin production. Thus, ultimately altering the habitat of resident gut bacteria and promoting changes in the gut microbiota composition (Gagne et al., 2013). Therefore, various feeding strategies have been developed in order to keep a suitable function of the GIT tract. Zeolite and in particular CZ have shown efficacy in animals (such as pigs, calves) and humans suffering from gastrointestinal disturbances (RodriguezFuentes et al., 1997; Papaioannou et al., 2005).

In this study, 20 adult English Setter dogs were trained and fed with a diet supplemented with CZ to evaluate how the microbiota and in particular bifidobacterial population as well as specific gut pathogens, could be modulated.

The results obtained after 29 days of CZ diet, showed that CZ affects the fecal microbial concentration but not the fecal score, which remained in a desirable range (well-formed, soft stools) for healthy dogs (Gagne et al., 2013). Notably, we observed an increase in relative abundance of *Lactobacillus* ssp. as well as *Bifidobacterium* ssp. phylotypes, accompanied by a decrease in phylotypes belonging to *Enterobacteriaceae* family in CZ fecal samples. This could be supported by the adsorptive capacity exploited by CZ toward *E. coli* and *C. perfringens*. Furthermore, *E. coli* and *Enterobacter* are common causes of extra-intestinal opportunistic infections in in dogs (Ogeer-Gyles et al., 2006), while *C. perfringens* is strongly related to hemorrhagic gastroenteritis (Schlegel et al., 2012).

Moreover, the major presence of lactobacilli and bifidobacteria could be very interesting since these bacterial taxa are considered to exploit beneficial roles on the health of their hosts (Gibson et al., 2005). In this context, various members of *Lactobacillus* and *Bifidobacterium* species are the most exploited probiotic bacteria utilized for pet (Kelley et al., 2010; Strompfova et al., 2014) and some of them have been suggested to improve the health and brain function of dogs (Biagi et al., 2007; Bravo et al., 2011). Increased concentrations of these microorganisms have been associated with decreased fecal concentrations of potentially pathogenic bacteria and decreased levels of carcinogenic and putrefactive compounds in digesta (Grieshop et al., 2002).

This is the first study where the bifidobacterial community of healthy dog was explored through a Next Generation Sequencing approach involving bifidobacterial ITS profiling. The obtained results allowed the identification of a bifidobacterial profile in English setter hunting dogs and revealed the presence of typical animal bifidobacteria such as *B. animalis* and *B. pseudolongum* and many putative new taxa. CZ treatment led to an increase of the abundance of *B. animalis* and *B. pseudolongum* species, which are characterized by the presence of genes encoding for exopolysaccharides structures that could lead to a special cell protection (Ferrario et al., 2016; Hidalgo-Cantabrana et al., 2016). Increase of the bifidobacterial strains coupled with the adsorptive capacity of CZ could bring to a reduction of species belonging to the *Enterobacteriaceae* family, such as *Klebsiella* and *Enterobacter*, typical dog pathogens (Gibson et al., 2008). Combined CZ treatment with probiotic supplementation, such as bifidobacterial strains, might enhance the reduction of canine pathogens as well as strength the beneficial effects on the animal health.

## Conclusion

Dietary CZ supplementation can help to maintain a balanced intestinal microbial ecosystem and to prevent stress-related GIT upsets in healthy dogs, with a decrease of gut pathogens and a remarkable increase of bifidobacteria. This is particularly relevant in training hunting dogs where the mental and physical stress, to which they are subjected during training periods, can affect GI permeability and motility. Further studies are needed to confirm the beneficially effect by CZ also in diseased dogs.

## Author Contributions

AS, PS, VB, and MO designed and performed experiments. MO, LM, AS, PS, and CF wrote the manuscript. LM and CM performed bioinformatic analyses. AS, CF, PS, MO, and VB performed experiments. CM, LM, ER, and FDI commented the manuscript. PS, AS, and MO conceived the study, revised and approved the manuscript. All authors reviewed the manuscript.

## Conflict of Interest Statement

The authors declare that the research was conducted in the absence of any commercial or financial relationships that could be construed as a potential conflict of interest.

The reviewer FB and handling Editor declared their shared affiliation, and the handling Editor states that the process nevertheless met the standards of a fair and objective review.

## Footnotes

1 http://probiogenomics.unipr.it/sw/bif_ITS_analysis.zip
