# Supplementary material for: Modulation of the Bifidobacterial Communities of the Dog Microbiota by Zeolite
Source: Front Microbiol. 2016 Sep 22;7:1491. doi: 10.3389/fmicb.2016.01491 (PMC5031887; doi:10.3389/fmicb.2016.01491)
Supplement: Supplementary file 1 [file Table_1.DOCX]

**Modulation of the bifidobacterial communities of the dog microbiota by zeolite**

Alberto Sabbioni, Chiara Ferrario, Christian Milani, Leonardo Mancabelli, Enzo Riccardi, Francesco Di Ianni, Valentino Beretti, Paola Superchi, Maria Cristina Ossiprandi

**Supplementary Materials**

**SupplementaryTable S1.** 16S rRNA microbial profiling data.

| **Sample** | **Number of sequenced pe reads** | **Number of pe reads with mean quality > 20** | **Number of merged pe reads** | **Human sequences** | **Length outside bounds of 100 and 400** | **Ambiguous bases** | **Homopolymers > 7** | **Mismatch in primers >1** | **Reverse primer not found** | **Final Read Number** |
| --- | --- | --- | --- | --- | --- | --- | --- | --- | --- | --- |
| T0 | 108804 | 105807 | 101447 | 443 | 0 | 0 | 5 | 1996 | 31 | 98972 |
| NTrT1 | 102499 | 97684 | 91921 | 236 | 0 | 0 | 16 | 1860 | 44 | 89765 |
| NTrT2 | 97943 | 92795 | 87242 | 240 | 0 | 0 | 9 | 1755 | 39 | 85198 |
| TrT1 | 95084 | 91687 | 87435 | 132 | 0 | 0 | 9 | 1740 | 34 | 85520 |
| TrT2 | 98686 | 93981 | 89766 | 282 | 0 | 0 | 11 | 1733 | 44 | 87696 |
