# Supplementary material for: Modulation of the Bifidobacterial Communities of the Dog Microbiota by Zeolite
Source: Front Microbiol. 2016 Sep 22;7:1491. doi: 10.3389/fmicb.2016.01491 (PMC5031887; doi:10.3389/fmicb.2016.01491)
Supplement: Supplementary file 2 [file Table_2.DOCX]

**Modulation of the bifidobacterial communities of the dog microbiota by zeolite**

Alberto Sabbioni, Chiara Ferrario, Christian Milani, Leonardo Mancabelli, Enzo Riccardi, Francesco Di Ianni, Valentino Beretti, Paola Superchi, Maria Cristina Ossiprandi

**Supplementary Materials**

**Supplementary Table S2.** ITS bifidobacterial profiling data.

| **Sample** | **Number of sequenced pe reads** | **Number of pe reads with mean quality > 20** | **Number of merged pe reads** | **Human sequences** | **Length outside bounds of 100 and 400** | **Ambiguous bases** | **Homopolymers > 7** | **Mismatch in primers >1** | | **Reverse primer not found** | **Final Read Number** |
| --- | --- | --- | --- | --- | --- | --- | --- | --- | --- | --- | --- |
| T0-ITS | 60745 | 59721 | 53315 | 0 | 0 | 0 | 4 | 787 | 493 | | 52031 |
| NTrT1-ITS | 63588 | 62290 | 56117 | 0 | 0 | 0 | 12 | 780 | 545 | | 54780 |
| NTrT2-ITS | 67211 | 65958 | 59469 | 0 | 0 | 0 | 6 | 937 | 488 | | 58038 |
| TrT1-ITS | 52754 | 51724 | 46269 | 0 | 0 | 0 | 9 | 647 | 364 | | 45249 |
| TrT2-ITS | 60109 | 58686 | 53475 | 0 | 0 | 0 | 8 | 715 | 508 | | 52244 |
